# Metabolic diversity and responses of anteater clostridial isolates to chitin-based substrates

**DOI:** 10.3389/fvets.2025.1459378

**Published:** 2025-02-19

**Authors:** Ahmad Amin, Nikol Modrackova, Vaclav Tejnecky, Vera Neuzil-Bunesova

**Affiliations:** ^1^Department of Microbiology, Nutrition and Dietetics, Czech University of Life Sciences Prague, Prague, Czechia; ^2^Department of Soil Science and Soil Protection, Czech University of Life Sciences Prague, Prague, Czechia

**Keywords:** clostridia, chitin, chitosan, *N*-acetyl-D-glucosamine, cellulose, fermentation, metabolites, antimicrobial activity

## Abstract

**Aim:**

*Clostridium* species, such as *Clostridium perfringens*, *C. baratii, C. colicanis, Paraclostridium bifermentans*, and *Paeniclostridium sordellii*, are Gram-positive, anaerobic, endospore-forming bacteria with diverse pathogenic mechanisms. While these species are commensals in the guts of variable animal species, such as anteaters, they are less frequently found in humans. The diet of anteaters, which includes chitin and formic acid, plays an important role in their specific dietary habits, as well as in clostridial metabolism.

**Methods and results:**

This study investigates the metabolic diversity and responses of anteater clostridial isolates to various substrates, namely chitin, chitosan, cellulose, *N*-acetyl-D-glucosamine (NAG), and glucose. All tested clostridia were able to grow in the presence of chitin, cellulose, NAG, and glucose, but varied in metabolite production. However, the presence of chitosan surprisingly showed an antimicrobial effect against clostridia, especially *Pae. sordellii*, *P. bifermentans,* and *C. colicanis*. The results demonstrate significant variations in fermentation profiles, and metabolite production across substrates and clostridial species. Acetate production was detected as common for all tested clostridia despite species variability and incoming substrates, as well as lactate, butyrate, propionate, and formate for some strains.

**Conclusion:**

In relation to digestion, anteater clostridia could play an important role in chitin and its degradation products, which, in the end, can influence clostridial occurrence and pathogenicity via chitosan.

## 1 Introduction

Clostridia, a diverse group of Gram-positive bacteria, play dual roles in the host gut as both pathogenic and commensal microorganisms. These bacteria are characterized by their ability to form endospores, which enable them to survive in diverse environments and withstand various forms of stress (1, 2). Pathogenic clostridial strains can cause severe diarrhea and colitis, particularly after hospitalization connected to antibiotic treatments, and can produce toxins during overgrowth or entering the bloodstream as well. Their presence can lead to gas gangrene, tetanus, and botulism outbreaks (1). In contrast to clostridial pathogenic counterparts, commensal strains play a crucial role in maintaining gut health. These bacteria constitute a significant proportion of the total intestinal microbiota, with the most abundant species belonging to *Clostridium* clusters IV and XIVa (1, 3, 4). Commensal clostridia establish a symbiotic relationship with the host, colonizing the intestinal mucosa and interacting with intestinal cells. Their multifaceted functions include modulating immune system activity and metabolic processes within the gut and preventing dysbiosis (1, 5). Additionally, they synthesize short-chain fatty acids (SCFAs), such as butyrate, which serve as an energy source for gut epithelial cells and exhibit anti-inflammatory effects (6, 7). Furthermore, commensal clostridia outcompete harmful pathogens for nutrients and adhesion sites, contributing to overall gut homeostasis (3).

In animals, diet plays a crucial role in determining the composition of the gut microbiota, favoring specific microbial populations based on dietary preferences (8), and significantly influencing the clostridial occurrence and function in host organisms. For instance, in termites, the class Clostridia forms a significant part of their gut microbiota, aiding in lignocellulose digestion and enabling survival on nitrogen-poor diets (9, 10). In cows, they are commonly present in the gut, contributing to overall gut health, although specific species can also cause disease under certain conditions (11). *Clostridium* species play vital roles in the anteater microbiome as well. Among these, *C. perfringens, C. baratii, C. colicanis, Paraclostridium bifermentans,* and *Paeniclostridium sordellii* were found in the fecal samples of Southern Tamanduas (*Tamandua tetradactyla*) in captive conditions (12). The occurrence of the mentioned clostridial species appears to correspond to the animal diet, the method of obtaining it, and the habits of anteaters. Specifically, the occurrence of *C. perfringens* is associated with a variety of environments, including soil, food, and sewage, and this species is a common member of the gastrointestinal microbiota of both diseased and non-diseased humans and animals. This corresponds to the highly variable phenotypic characteristics of individual strains, which is common for multi-host species (13, 14) belonging to the *Clostridiaceae* family. Similarly, *C. baratii* strains (*Lachnospiraceae* family) have been isolated from food, soil, and animal and human fecal samples (15–17). They are rare opportunistic pathogens associated with botulism intoxication carried asymptomatically or causing botulism outbreaks. Interestingly, this species is not taxonomically related to *C. botulinum*, but some strains are equipped with a BoNT/F7 cluster (16). Then, *C. colicanis* belonging to *Lachnospiraceae* family as well, which was first isolated from dog feces, might be part of the normal gut microbiota of dogs (18). Similarly, *C. baratii* has been also previously isolated from feces, namely marmoset feces (15) and *Pae. sordellii* has been isolated from the dog fecal microbiota together with *C. perfringens* (19). *Pae. sordellii* occurrence is typical for human gut microbiota as well (17). The majority of strains are non-pathogenic, but some have been associated with severe infections in humans and animals. In humans, *Pae. sordellii* is mainly associated with trauma, toxic shock, soft tissue skin, and gynecological infections (20). Genomic analysis of a pathogenic strain *Pae. sordellii* CBA7122 identified several known virulence factors in the genome (21). Furthermore, *P. bifermentans* is an emerging human pathogen that is phylogenomically close to *Pae. sordellii*, both belonging to the *Peptostreptococcaceae* family. Moreover, *P. bifermentans* can produce endospores and various toxins, and it has been associated with food poisoning, and wound and uterine infections in animals. Furthermore, it can ferment different carbohydrates and produce foul-smelling compounds (22).

Research by Takahashi et al. (10) highlights clostridial contribution to food material degradation and the production of compounds that maintain immune homeostasis within the gut environment. The anteater diet primarily consists of ants and termites and may occasionally consume other insects or insect larvae. This diet is a common source of chitin and its derivatives. Furthermore, as hydrochloric acid is not produced in anteaters’ stomachs like in most mammals, they depend on the supply of formic acid from swallowed ants for their digestion (23). The anteater diet in captive conditions is usually supplemented with chitin and formic acid (12). Anteaters live in mutually beneficial symbiosis with the gut microbiota that helps them break down chitin, cellulose, and other complex carbohydrates (24). The role of clostridial strains in the microbiota of anteaters is not clear. Therefore, this study investigates the metabolic diversity and responses of various anteater clostridial isolates to various substrates, specifically their growth and metabolite production on chitin, chitosan, cellulose, *N*-acetyl-D-glucosamine (NAG), and glucose as a control.

## 2 Materials and methods

### 2.1 Strains origin, manipulation, and identification

Clostridial strains (*n* = 22) belonging to the species *C. perfringens* (*n* = 7), *C. baratii* (*n* = 4), *C. colicanis* (*n* = 5), *P. bifermentans* (*n* = 4), and *Pae. sordellii* (*n* = 2) were obtained from the fecal samples of Southern Tamanduas (*Tamandua tetradactyla*) previously analyzed by Amin et al. (12) and were deposited in the strain collection of the Department of Microbiology, Nutrition, and Dietetics (CZU, Czechia). Bacterial culture stocks were stored at − 20°C in 30% glycerol (VWR, USA) and at room temperature in Cooked Meat Medium (CMM; Thermo Fisher Scientific, USA), respectively. To obtain a working culture, a clostridial cryostock was always streaked on the Wilkins-Chalgren Anaerobe Agar supplemented with 5 g L^−1^ of GMO-Free Soya Peptone (both Oxoid, UK), 0.5 g L^−1^ of L-cysteine, and 1 mL L^−1^ of Tween 80 (both Sigma-Aldrich, USA) (WSP agar) and was cultivated at 37°C for 48 h under anaerobic conditions using GENbag anaer (BioMérieux, France). Then, a clostridial colony was isolated to the anaerobic tubes (25) filled with WSP broth and cultivated at 37°C for 24 h. Then, the cell morphology and culture purity were determined by phase-contrast microscopy (Nikon Eclipse E200, Japan), and strain identity was evaluated using MALDI-TOF MS with ethanol-formic acid extraction procedure with HCCA matrix to the species level using Biotyper software (server distribution version 4.1.100 (PYTH), build 174; server module version 4.3.18, build 330) according to the manufacturer’s instructions (Bruker Daltonik GmbH, Germany) and Modrackova et al. (26). Then, the identity of the strains was further verified by 16S rRNA sequencing. Briefly, bacterial DNA was isolated using PrepMan Ultra™ (Applied Biosystems, USA) according to the manufacturer’s instructions and stored at −20°C. Primers fd1 (5′-AGAGTTTGATCCTGGCTCAG-3′) and rp2 (5′ ACGGCTACCTTGTTACGACTT-3′) were used to amplify the 16S rRNA gene via PCR reaction according to Weisburg et al. (27). Then, PCR products were purified with E.Z.N.A. Cycle Pure Kit (Omega Bio-Tek, USA) and were Sanger sequenced by Eurofins Genomics (Germany). The obtained sequences were processed in Chromas Lite 2.5.1 (Technelysium Pty Ltd., Australia) and BioEdit (28) using the ClustalW algorithm (29) with subsequent classification using the EzBioCloud database (30). Moreover, to obtain strain fingerprint profiles, (GTG5) primer (5′-GTGGTGGTGGTGGTG-3′) for repetitive element sequence-based REP-PCR was used according to Masco et al. (31) and Bunesova et al. (32). Similarities between strains were determined by UPGMA analysis using BioNumerics (BioMérieux). Based on the above identifications, six clostridial strains were selected for further testing—one strain from each species and two strains from *C. colicanis* because of their different cell morphologies.

### 2.2 Phenotypic characterization of selected strains

Selected strains (*n* = 6; see section 2.1) were characterized for specific enzymatic and fermentation activity. All experimental studies were performed with freshly grown working cultures and in at least independent duplicates. Fermentation profiles of tested strains were obtained using ANAEROTEST 23 (Erba Lachema, Czechia) and API 20A (BioMérieux). Both biochemical tests were performed and evaluated according to the manufacturers’ instructions after anaerobic cultivation (GENbag anaer) at 37°C for 48 h. The color change from purple to yellow indicated a metabolic activity (positive reaction). Some unclear reactions needed to be verified by adding bromocresol purple solution (BCP; BioMérieux), as instructed by the manufacturer for the API 20A kit.

### 2.3 Clostridial ability to grow in the presence of chitin-based substrates as a single source of energy

The ability of clostridial strains to grow and produce metabolites in the presence of chitin, chitosan, NAG (all Sigma-Aldrich), cellulose (SERVA, Germany), glucose (Lachner, Czechia) as a positive control, and API 20A medium (BioMérieux) as a negative control was investigated. First, the API 20A medium was prepared as a basic medium: 5 g L^−1^ of trypticase, 5 g L^−1^ of yeast extract (both Oxoid), 2.5 g L^−1^ of sodium chloride (Lachner), 0.2 g L^−1^ of L-tryptophan, 0.4 g L^−1^ of L-Cysteine, 0.005 g L^−1^ of hemin (all Sigma-Aldrich), 0.01 g L-1 of vitamin K1 (Thermo Fisher Scientific), 0.1 g L^−1^ of sodium sulfite (Sigma-Aldrich), and 0.17 g L^−1^ of bromocresol purple (Sigma-Aldrich). The pH was adjusted to the range of 6.9–7.3. Then, the medium was supplemented with 2 g L^− 1^ of the mentioned substrates, distributed to the tubes, treated with CO_2_ (25), and sterilized by autoclaving. For clostridial inoculation, overnight working cultures were centrifuged, and pellets were re-suspended in the same volume of API 20A medium. Then, the media were inoculated with clostridial culture (0.5% total inoculum concentration) with subsequent anaerobic incubation at 37°C for 48 h. Moreover, metabolic products were quantified, as well as pH values of each tested variant were measured using Checker Plus-pH HI98100 (Hanna instruments, Czechia). Every strain was tested at least two times.

#### 2.3.1 Measurement of short-chain fatty acid and lactate levels

To determine the main clostridial metabolites (lactate, acetate, butyrate, formate, and propionate), capillary high-pressure ion-exchange chromatography with suppressed conductivity detection (IC-SC) was used. Specifically, they were measured in clostridial supernatants from API 20A media with chitin-based substrates after 48 h of anaerobic cultivation at 37°C. Supernatants were diluted (500×) and filtered through a 0.45-μm nylon membrane and analyzed using a Dionex ICS 4000 system equipped with IonPac AS11-HC 4 μm guard and analytical columns (Thermo Scientific). Eluent composition was as follows: 0–10 min isocratic: 1 mM KOH; 10–20 min linear gradient: 1–60 mM KOH; and 20–25 min again isocratic: 60 mM KOH. The flow rate was set to 0.012 mL min^−1^. An ACES 300 suppressor (Thermo Scientific) was used to suppress eluent conductivity, while a carbonate Removal Device 200 (Thermo Scientific) was implemented to suppress carbon dioxide baseline shift. Chromatograms were processed with Chromeleon 7.20 (Thermo Fisher). Standards were prepared from 1 g L^−1^ of stock solutions (Analytika, Czechia; Inorganic Ventures, USA) and diluted to range from 0.1 to 40 mg L^−1^. Deionized water (conductivity <0.055 μS cm^−1^) was used for eluent and standard preparation. Similarly, to get metabolite profiles on nutrition-rich medium, clostridial supernatants from freshly grown cultures in WSP broth after 24 h anaerobic cultivation at 37°C were measured as well. All measurements were performed in duplicates.

### 2.4 Antimicrobial effect of chitosan on clostridial growth

The ability of clostridial strains to grow and survive in the presence of chitosan in concentration 2 g L^−1^, which was used in previous tests was evaluated. The test was performed in WSP broth and API 20A medium. Media preparation is described above, with API 20A media prepared without bromocresol purple. In both variants, clostridia were cultured simultaneously in a medium with chitosan (2 g L^−1^) and a medium without it. The media were inoculated with clostridial culture (0.5% total inoculum concentration as before) with subsequent anaerobic incubation at 37°C. Testing was conducted in two repetitions, and bacterial counts in each of the variants were determined by cultivation on WSP agar at the time 0, after 24 and 48 h. All the plates were incubated anaerobically using GENbag anaer (bioMérieux) at 37°C for 2 days. Then, the cultivation counts (log CFU mL^−1^) were determined.

## 3 Results

### 3.1 Identity and biochemical characteristics of selected anteaters’ clostridial strains

Clostridial strains were isolated from fecal samples of Southern Tamanduas and identified in previous studies (12) by the MALDI-TOF MS method. From a total of 22 strains belonging to 5 species, 6 strains were selected for further characterization and testing based on morphology, fingerprint REP-PCR profiles, and identification of the 16S rRNA gene. Six different morphologies were recorded for the five tested species identified by MALDI-TOF MS as *C. perfringens*, *C. baratii, C. colicanis, P. bifermentans*, and *Pae. sordellii*. When strains identified as *C. colicanis* had two different morphologies, they were short (S) or long (L) regular bacilli. The REP-PCR method allowed differentiation of the five identified species, but strain differences were not noted, even for *C. colicanis* with different morphology. Although the strains came from different anteater individuals, they appeared as identical copies, which was also confirmed by the identification of the 16S rRNA gene. The results of the identification of the six selected strains are shown in Table 1. The 16S rRNA gene identification, even though it was a relatively long section (1411–1,435 bp), was not sufficient to confirm the MALDI-TOF MS identification of *C. baratii* 135_1 and *P. bifermentans* 139_1. For *C. perfringens* A.E.T 8, *C. colicanis* strain 132_1 and 95B_14, and *Pae. sordellii* 85A_1, the identification was confirmed, and the matches with other taxa were less than 98.5%, respectively. The partial 16S rRNA gene sequences were uploaded to the NCBI database. Their accession numbers are listed in Table 1.

**Table 1:** Identification of selected clostridial strains.

Strain	MALDI-TOF MS	bp	16S rRNA gene identification	Similarity	Accession number
135_1	*C. baratii*	1,415	*C. baratii*	99.86%	PQ573793
			*C. budayi*	99.65%	
			*C. nitritogenes*	99.36%	
			*C. sardiniense*	98.94%	
131_2 (L)	*C. colicanis*	1,431	*C. colicanis*	99.86%	PQ573580
95B_14 (S)	*C. colicanis*	1,431	*C. colicanis*	99.86%	PQ573579
A.E.T 8	*C. perfringens*	1,429	*C. perfringens*	99.86%	PQ573794
139_1	*P. bifermentans*	1,411	*P. dentum*	99.93%	PQ573795
			*P. benzoelyticum*	99.86%	
			*P. bifermentans* subsp. *muricolitidis*	99.78%	
			*P. bifermentans* subsp. *bifermentans*	99.57%	
85A_1	*Pae. sordellii*	1,415	*Pae. sordellii*	99.64%	PQ573511

Primers fd1 and rp2 were used to amplify the 16S rRNA gene through PCR reaction, and classification of edited sequences was conducted using the EzBioCloud database.

Fermentation profiles of the six tested clostridial strains were obtained using ANAEROTEST 23 and API 20A. The recorded profiles were identical, so one common table was created from the tests (Table 2). *C. perfringens* A.E.T 8, *C. baratii* 135_1, and *P. bifermentans* 139_1 had the largest number of positive reactions on saccharide substrates. Positive enzymatic activity of *N*-acetyl-*β*-D-glucosaminidase and β-glucosidase was also detected in them, and they had a positive esculin test. Both strains of *C. perfringens* and *P. bifermentans* also were positive on bovine-origin gelatin. Whereas *C. baratii* was positive for the nitrate test along with both *C. colicanis* strains 132_1 and 95B_14. These strains, with the same identity based on MALDI-TOF MS and 16S r DNA, had different morphologies and their fermentation profiles also differed. Although the *C. colicanis* 95B_14 strain did not utilize any of the tested saccharide substrates to form acids, it nevertheless tested positive for *N*-acetyl-*β*-D-glucosaminidase, β-glucosidase, and esculin. In addition to the positive reaction for nitrates mentioned above, this strain was also positive for urease. The second strain, *C. colicanis* 132_1, was able to utilize only four substrates, namely glucose, fructose, maltose, and partially sorbitol. *Pae. sordellii* also had a similar profile, which fermented only easily fermented substrates such as glucose, fructose, and maltose, and had a positive result for indole, as well as *P. bifermentans* and *C. baratii.*

**Table 2:** Fermentation profiles of clostridial strains using ANAEROTEST 23 and API 20A tests.

	Glucose	Fructose	Galactose	Rhamnose	Mannose	Xylose	Arabinose	Mannitol	Sorbitol	Maltose	Lactose	Sucrose/D-saccharose	Trehalose	Cellobiose	Raffinose	Melezitose	Salicin	N-acetyl-β-D-glucosaminidase	β-glucosidase	Esculin	Gelatine (bovine-origin)	Nitrate	Urease	Indole	Glycerol
*C. baratii* 135_1	+	+	−	−	+	−	−	−	−	+/−	+/−	+	−	+	−	−	+	+	+	+	−	+	−	+	+/−
*C. colicanis* (L) 131_2	+	+	−	−	−	−	−	−	+/−	+	−	−	−	−	−	−	−	−	−	−	−	+	−	−	−
*C. colicanis* (S) 95B_14	−	−	−	−	−	−	−	−	−	−	−	−	−	−	−	−	−	+	+	+	−	+	+	−	−
*C. perfringens* A.E.T. 8	+	+	+	−	+	−	−	+/−	−	+	+	+	+	−	+	−	−	+	+	+	+	−	−	−	+
*P. bifermentans* 139_1	+	−	−	−	+	−	−	−	−	+	−	−	−	−	−	−	+	+	+	+	+	−	−	+	−
*Pae. sordellii* 85A_1	+	+	−	−	−	−	−	−	−	+	−	−	−	−	−	−	−	−	−	−	−	−	−	+	−

The biochemical tests were prepared according to the manufacturer’s instructions. The results were read after 48 h; (+) positive reaction, (+/−) slightly positive, (−) negative reaction. Some reactions had to be stained with BCP, as the color indicator, because it was degraded after cultivation and the given well appeared colorless. Substrates that needed to be repainted with BCP are marked in gray.

The main metabolite products of clostridial strains after cultivation in the nutrition-rich medium are shown in Table 3. Acetate, lactate, formate, and butyrate were detected as the main metabolism products of tested clostridial strains in the presence of available and easily fermented substrates (glucose and soya peptone). Clostridial ability to produce metabolites was strain-specific. Specifically, *P. bifermentans* 137_1, *C. colicanis* 131_2, and *Pae. sordellii* 85A_1 produced the highest levels of acetate, while *C. baratii* 135_1 and *C. colicanis* 131_2 lactate, *P. bifermentans* 139_1, *C. colicanis* 131_2, *Pae. sordellii* 85A_1, and *C. colicanis* 95B_14 formate, and *C. colicanis* 131_2, *C. colicanis* 95B_14, and *C. perfringens* A.E.T 8 butyrate. Slightly increased levels of propionate were detected for *C. perfringens* A.E.T 8, *P. bifermentans* 139_1, and *Pae. sordellii* 85A_1. When comparing two strains of *C. colicanis* with different cell morphology, they exhibited various manners in metabolite production.

**Table 3:** Concentration of metabolites (mM) after clostridial cultivation in WSP broth.

Strain	Medium	Lactate	Acetate	Propionate	Formate	Butyrate
*C. baratii* 135_1	WSP	**52.42 ± 3.20**	15.79 ± 4.89	0.34 ± 0.16	0.44 ± 0.30	2.38 ± 0.96
*C. colicanis* (L) 131_2	WSP	**23.79 ± 3.13**	**27.45 ± 0.19**	0.46 ± 0.06	**15.03 ± 1.32**	**16.05 ± 1.92**
*C. colicanis* (S) 95B_14	WSP	7.37 ± 1.52	10.91 ± 2.32	0.35 ± 0.11	**9.00 ± 0.79**	**11.21 ± 1.64**
*C. perfringens* A.E.T 8	WSP	5.29 ± 1.24	16.22 ± 2.22	**1.66 ± 0.67**	1.33 ± 0.28	**6.54 ± 2.01**
*P. bifermentans* 139_1	WSP	**5.53 ± 2.91**	**32.41 ± 2.81**	**1.80 ± 0.60**	**20.96 ± 1.65**	**0.82 ± 0.23**
*Pae. sordellii* 85A_1	WSP	2.93 ± 3.45	**21.39 ± 0.47**	**1.55 ± 0.73**	**12.07 ± 4.83**	0.53 ± 0.26

Clostridial strains were anaerobically cultivated in WSP broth at 37°C for 24 h. Then, the produced metabolites were measured by capillary high-pressure ion exchange chromatography with suppressed conductivity detection (IC-SC). Considerably higher concentration levels are marked as bold text. WSP, Wilkins-Chalgren Anaerobe supplemented with soya peptone, L-cysteine, and Tween 80.

### 3.2 Growth and metabolite formation on chitin-based substrates

The ability of clostridial strains to grow and produce metabolites in the presence of chitin, chitosan, cellulose, NAG, and glucose is shown in Table 4. The detection of main clostridial metabolites was influenced mainly by the presence of substrate added to API 20A medium and by the tested strain of clostridia as well. *P. bifermentans* 137_1 exhibited the highest fermentation ability to use all given substrates except chitosan. Acetate was its main metabolite product, followed by propionate and formate. Surprisingly, negative control served as a better growth substrate compared to glucose, where the propionate was not produced. Then, *C. perfringens* A.E.T 8 mainly used NAG followed by chitin, cellulose, and glucose with considerable acetate and butyrate production. It used the negative control as well but with exclusive acetate production. *Pae. sordellii* 85A_1 utilized chitin, cellulose, NAG, and glucose with acetate and formate as the main metabolite products, followed by slightly increased propionate levels except for glucose. *C. colicanis* 95B_14 used NAG and glucose with the main production of acetate, butyrate, and lactate. It then utilized chitin and negative control with acetate, lactate, formate, and butyrate production except for the butyrate on the API 20A medium. Interestingly, *C. colicanis* 131_2 was less competent in the utilization of provided substrates. Specifically, it used NAG and glucose followed by the production of acetate, lactate, and butyrate, and probably partially chitin as well, with a slight increase in butyrate level. *C. baratii* 135_1 mostly used NAG with the production of acetate, lactate, and butyrate, as well as chitin and cellulose with acetate production, and glucose with lactate, acetate, and butyrate production. The average pH value after the clostridial cultivation was 6.56 ± 0.15 for API 20A medium, 5.68 ± 0.36 for glucose, 6.50 ± 0.06 for chitin, 6.83 ± 0.09 for chitosan, 6.50 ± 0.07 for cellulose, and 6.03 ± 0.13 for NAG. If acetate and lactate were produced, the pH values decreased accordingly.

**Table 4:** Concentration of metabolites (mM) after clostridial cultivation in the API 20A medium with various substrates.

Strain	Substrate	pH	Lactate	Acetate	Propionate	Formate	Butyrate
*C. baratii* 135_1	API 20A (−)	6,72	0.09 ± 0.06	0.25 ± 0.09	0.06 ± 0.05	0.06 ± 0.02	0.03 ± 0.03
	Glucose (+)	5,27	**19.80 ± 1.18**	**3.72 ± 0.19**	0.06 ± 0.02	0.08 ± 0.01	**1.78 ± 0.12**
	Chitin	6,52	0.52 ± 0.13	**3.04 ± 0.19**	0.12 ± 0.02	0.06 ± 0.03	0.34 ± 0.02
	Chitosan	6,77	0.10 ± 0.05	0.23 ± 0.09	0.05 ± 0.06	0.06 ± 0.03	0.02 ± 0.04
	Cellulose	6,55	0.54 ± 0.23	**2.46 ± 0.35**	0.11 ± 0.00	0.10 ± 0.03	0.24 ± 0.04
	NAG	5,86	**7.81 ± 0.13**	**8.17 ± 0.47**	0.09 ± 0.02	0.28 ± 0.01	**1.07 ± 0.01**
*C. colicanis* (L) 131_2	API 20A (−)	6,35	0.48 ± 0.15	0.83 ± 0.13	0.08 ± 0.04	0.27 ± 0.04	0.98 ± 0.07
	Glucose (+)	6,25	**5.55 ± 0.51**	**2.32 ± 0.08**	−0.02 ± 0.03	0.41 ± 0.07	**3.92 ± 0.07**
	Chitin	6,46	0.45 ± 0.07	0.93 ± 0.11	0.13 ± 0.02	0.79 ± 0.09	**1.30 ± 0.03**
	Chitosan	6,78	−0.06 ± 0.03	0.03 ± 0.12	−0.04 ± 0.02	0.17 ± 0.04	0.49 ± 0.06
	Cellulose	6,51	0.18 ± 0.04	0.45 ± 0.05	0.11 ± 0.10	0.81 ± 1.04	0.00 ± 0.04
	NAG	6,07	**1.97 ± 0.23**	**6.70 ± 0.10**	0.00 ± 0.07	0.76 ± 0.06	**3.44 ± 0.03**
*C. colicanis* (S) 95B_14	API 20A (−)	6,67	**3.80 ± 1.02**	**2.01 ± 0.78**	0.08 ± 0.09	**1.01 ± 0.19**	0.78 ± 0.24
	Glucose (+)	5,86	**1.06 ± 0.46**	**2.60 ± 1.00**	0.04 ± 0.07	0.87 ± 0.13	**2.97 ± 0.88**
	Chitin	6,51	**2.45 ± 0.37**	**2.86 ± 0.71**	0.08 ± 0.07	**1.25 ± 0.16**	**1.09 ± 0.21**
	Chitosan	6,73	0.10 ± 0.12	0.50 ± 0.38	0.10 ± 0.11	0.31 ± 0.08	0.73 ± 0.19
	Cellulose	6,54	0.18 ± 0.20	0.59 ± 0.47	0.09 ± 0.10	0.25 ± 0.11	0.71 ± 0.25
	NAG	6,05	**3.56 ± 0.73**	**7.85 ± 1.63**	0.07 ± 0.05	0.67 ± 0.10	**3.48 ± 0.65**
*C. perfringens* A.E.T. 8	API 20A (−)	6,42	−0.01 ± 0.16	**3.67 ± 0.69**	0.53 ± 0.12	0.33 ± 0.01	0.90 ± 0.23
	Glucose (+)	5,80	0.82 ± 0.09	**12.71 ± 0.69**	0.83 ± 0.03	0.54 ± 0.02.	**6.96 ± 0.12**
	Chitin	6,48	0.13 ± 0.36	**4.28 ± 1.78**	0.67 ± 0.33	0.21 ± 0.13	**1.15 ± 0.58**
	Chitosan	6,98	0.14 ± 0.36	0.57 ± 0.49	0.06 ± 0.02	0.08 ± 0.06	0.17 ± 0.10
	Cellulose	6,54	0.23 ± 0.07	**5.18 ± 0.24**	0.84 ± 0.01	0.28 ± 0.04	**1.27 ± 0.02**
	NAG	5,99	0.27 ± 0.15	**15.06 ± 1.45**	0.89 ± 0.08	0.48 ± 0.01	**5.82 ± 0.38**
*P. bifermentans* 139_1	API 20A (−)	6,53	0.32 ± 0.23	**11.40 ± 2.12**	**6.68 ± 1.52**	**7.30 ± 1.97**	0.43 ± 0.14
	Glucose (+)	5,42	0.11 ± 0.10	**13.19 ± 1.19**	0.52 ± 0.07	**10.73 ± 1.08**	0.32 ± 0.03
	Chitin	6,61	0.21 ± 0.25	**11.20 ± 2.50**	**5.71 ± 1.55**	**7.00 ± 1.95**	0.32 ± 0.13
	Chitosan	6,84	0.15 ± 0.09	0.37 ± 0.02	0.09 ± 0.04	0.11 ± 0.02	0.05 ± 0.03
	Cellulose	6,48	0.09 ± 0.08	**11.28 ± 1.27**	**6.00 ± 0.68**	**5.30 ± 0.47**	0.32 ± 0.03
	NAG	5,98	0.10 ± 0.08	**12.14 ± 1.18**	**2.18 ± 0.25**	**7.06 ± 0.67**	0.18 ± 0.03
*Pae. sordellii* 85A_1	API 20A (−)	6,68	−0.07 ± 0.01	0.19 ± 0.18	0.03 ± 0.04	0.10 ± 0.03	0.01 ± 0.03
	Glucose (+)	5,48	0.00 ± 0.01	**10.64 ± 0.11**	0.26 ± 0.01	**6.78 ± 0.03**	0.15 ± 0.02
	Chitin	6,44	−0.08 ± 0.04	**6.97 ± 0.33**	**1.17 ± 0.05**	**2.49 ± 0.06**	0.12 ± 0.01
	Chitosan	6,88	−0.01 ± 0.00	0.08 ± 0.18	0.02 ± 0.06	0.06 ± 0.00	0.01 ± 0.04
	Cellulose	6,36	−0.08 ± 0.03	**7.32 ± 0.25**	**1.42 ± 0.07**	**2.78 ± 0.02**	0.13 ± 0.02
	NAG	6,25	−0.05 ± 0.00	**5.99 ± 0.48**	**1.00 ± 0.01**	**2.84 ± 0.06**	0.11 ± 0.02

Clostridial strains were anaerobically cultivated in the API 20A medium with chitin-based substrates (chitosan, cellulose, NAG) at 37°C for 24 h. API 20A served as a negative control, while glucose was a positive control. The produced metabolites were measured in supernatants after the cultivation by capillary high-pressure ion-exchange chromatography with suppressed conductivity detection (IC-SC). Non-zero values are marked as bold text. NAG, *N*-acetyl-D-glucosamine.

### 3.3 Antimicrobial potential of chitosan on clostridial growth

The antimicrobial potential of chitosan in a WSP medium containing glucose and soya peptone is shown in Table 5. We found, the presence of chitosan did not negatively influence the growth of *C. baratii* 135_1 and *C. perfringens* A.E.T 8 after 24 h cultivation, while it did for *C. colicanis* 131_2, *P. bifermentans* 139_1, and *Pae. sordellii* 85A_1. Furthermore, the counts of *C. colicanis* 95B_14 considerably decreased after 48 h as well. The prolongated cultivation time caused a slight decrease of cultivation counts on a control medium of both *C. colicanis* strains and *P. bifermentans* 139_1, while the counts of other strains remained the same or even slightly increased.

**Table 5:** Antimicrobial potential of chitosan added to WSP broth against clostridia.

Strain	24 h			48 h		
	WSP	WSP + CHTS	Difference	WSP	WSP + CHTS	Difference
*C. baratii* 135_1	5.95 ± 1.12	6.77 ± 0.04	0.82	6.38 ± 0.06	5.53 ± 0.03	−0.85
*C. colicanis* (L) 131_2	**5.96 ± 1.34**	**2.87 ± 0.16**	**−3.09**	4.48 ± 0.05	2.90 ± 0.02	−0.58
*C. colicanis* (S) 95B_14	6.03 ± 0.81	5.18 ± 0.22	−0.85	**4.82 ± 0.03**	**2.50 ± 0.01**	**−2.32**
*C. perfringens* A.E.T. 8	7.01 ± 1.00	7.73 ± 0.02	0.72	7.20 ± 0.09	7.09 ± 0.08	−0.11
*P. bifermentans* 139_1	6.00 ± 1.28	4.27 ± 0.07	−1.73	5.16 ± 0.06	3.66 ± 0.00	−1.50
*Pae. sordellii* 85A_1	**6.08 ± 0.99**	**0**	**−6.08**	**6.03 ± 0.01**	**0**	**−6.03**

To assess the potential antimicrobial character of chitosan, clostridial strains were anaerobically cultivated in WSP broth with or without chitosan at 37°C for 24 and 48 h. Then, cultivation counts in log CFU mL^−1^ were determined and a difference between the test and control variants was calculated. A decrease in cultivation counts by more than 1.5 orders of magnitude is marked in bold. WSP, Wilkins-Chalgren Anaerobe broth supplemented with soya peptone, L-cysteine, and Tween 80; CHT, chitosan, WSP + CHTS, WSP broth with 2 g L^−1^ of chitosan.

Chitosan’s antimicrobial potential against clostridia was also tested in nutrient-poor API 20A medium (Table 6). The presence of chitosan did not negatively influence the growth of *C. baratii* 135_1 and *C. colicanis* 95B_14 after 24 h cultivation, while it did for *C. colicanis* 131_2, *P. bifermentans* 139_1, *P. sordellii* 85A_1, and slightly for *C. perfringens* A.E.T 8. A similar trend was confirmed after 48 h. Interestingly, the prolongated time increased the cultivation numbers of *C. baratii* 135_1, *B. colicanis* 131_2, and *C. colicanis* 95B_14 in control API 20A medium, while the counts of *C. perfringens* A.E.T 8, *P. bifermentans* 139_1, and *Pae. sordellii* 85A_1 decreased.

**Table 6:** Antimicrobial potential of chitosan added to API 20A medium against clostridia.

Strain	24 h			48 h		
	API20A	API20A + CHTS	Difference	API20A	API20A + CHTS	Difference
*C. baratii* 135_1	3.66 ± 0.11	5.37 ± 0.01	2.01	5.39 ± 0.01	5.21 ± 0.01	−0.19
*C. colicanis* (L) 131_2	**5.82 ± 0.04**	**2.88 ± 0.03**	**−2.94**	**6.44 ± 0.04**	**2.58 ± 0.05**	**−3.87**
*C. colicanis* (S) 95B_14	2.30 ± 0.00	3.74 ± 0.01	0.49	2.95 ± 0.07	3.44 ± 0.06	0.49
*C. perfringens* A.E.T. 8	7.61 ± 0.00	7.47 ± 0.01	−0.14	6.62 ± 0.03	5.97 ± 0.02	−0.65
*P. bifermentans* 139_1	**5.73 ± 0.01**	**4.45 ± 0.21**	**−1.28**	**4.59 ± 0.01**	**2.57 ± 0.02**	**−2.02**
*Pae. sordellii* 85A_1	**4.45 ± 0.21**	**0**	**−4.45**	**3.36 ± 0.02**	**0**	**−3.36**

To assess the potential antimicrobial character of chitosan, clostridial strains were anaerobically cultivated in the API 20A medium with or without chitosan at 37°C for 24 and 48 h. Then, cultivation counts in log CFU mL^−1^ were determined and a difference between the test and control variant was calculated. A decrease in cultivation counts by more than 1.5 orders of magnitude is marked in bold. CHT, chitosan, API 20A + CHTS, API 20A medium with 2 g L^−1^ of chitosan.

## 4 Discussion

The occurrence of clostridia is highly variable. We can find them in the infant and human gut microbiota (4, 17), or various animal hosts, they play an important role in the soil (33), and their occurrence is also linked to extreme conditions, which is linked to their ability to produce endospores (34). The five species identified as *C. perfringens*, *C. baratii*, *C. colicanis*, *P. bifermentans*, and *Pae. sordellii* tested and characterized in this article were isolated from fecal samples of anteaters, namely Southern Tamanduas (12). The class Clostridia includes a large and diverse number of taxa whose classification is often problematic (4). *C. baratii* is closely related to several species of *Eubacterium* spp., particularly *Eubacterium budayi* (syn. *Clostridium budayi*) (35). Therefore, the close similarity of *C. baratii* to *C. budayi* detected using 16S rRNA sequencing is not surprising. Similarly, there is known also close genetic similarity among *P. bifermentans*, *P. dentum*, and *P. benzoelyticum* (36, 37). Therefore, 16S rDNA sequencing was not sufficient to distinguish them and confirmed the identity of *P. bifermentans* 139_1 based on the MALDI-TOF MS. Furthermore, *Pae. sordellii* shows high genomic similarity to these species; however, similarity based on the 16S rRNA gene was less than 98%. The identity of the other clostridial species was consistent using the two mentioned methods used.

*C. perfringens* (*Clostridiaceae* family), *C. baratii*, *C. colicanis* (both *Lachnospiraceae* family), *P. bifermentans,* and *Pae. sordellii* (*Peptostreptococcaceae* family) were previously detected by Amin et al. (12) in several hosts of Southern Tamanduas. Moreover, they were from different breeding environments, so these clostridial species appear to be typical for the intestinal microbiota of anteaters. These families are also significantly represented in the feces of Sunda Pangolins, and their gut microbiome reveals its adaptation to specialized myrmecophagy (38). In mammals, ant and termite consumption represents a striking example of dietary convergence. This trait evolved independently at least five times in placentals with myrmecophagous species comprising aardvarks, anteaters, some armadillos, pangolins, and aardwolves (24). While clostridial species usually pose a pathogenic risk (39, 40), they can also contribute to the digestive process in animals such as anteaters. By utilizing nutrients that the host cannot directly digest, they produce SCFAs essential for intestinal homeostasis (1, 41, 42). The class Clostridia exhibits a great diversity and covers a broad metabolic and functional range, obtaining energy from different fermentative pathways that break down unused intestinal carbohydrates and proteins, yielding a variety of fermentation products, namely short-chain and branched-chain fatty acids, CO_2_, and H_2_ (43). The fermentation profiles of the tested clostridial species and strains were highly variable. From the ability to utilize simple or more complex carbohydrates to the activity of the enzyme *N*-acetyl-*β*-D-glucosaminidase, or β-glucosidase, which performs hydrolysis of various plant glycosides and oligosaccharides (44). The ability to utilize bovine-origin gelatine of some strains pointed to their proteolytic activity. Furthermore, among these isolates were indole-producing strains. The variability of the fermentation profiles of the two morphologically different strains, correctly identified using 16S rDNA as *C. colicanis*, was interesting, and further genomic characterization is desirable because the strain variability in clostridial species is known (45).

All characterized strains grew well in modified WSP agar, as was for clostridial species previously stated (17). Produced metabolites considerably varied among clostridial species. Acetate was produced similarly by all tested species, however, lactate production was dominated by *C. baratii*, butyrate by both *C. colicanis* strains, as well as formate. The latter was further significantly produced by *P. bifermentans* and *Pae. sordellii*. Formic acid is a naturally occurring compound found in ants and other insects. As a feed additive, formic acid enhances nutrient digestibility and utilization in animals while also lowering the pH of the digestive tract and inhibiting pathogen growth (46). For anteaters, formic acid assumes particular significance. Unlike many other animals, anteaters lack gastric acid secretion. Instead, they rely on consuming insects such as ants that contain formic acid. In essence, formic acid acts as a surrogate digestive acid for anteaters (47). Formic acid also acts as an antimicrobial agent within the anteaters’ digestive system, potentially aiding in the regulation of microbial populations (46).

Ants serve as a nutrient-rich food source for anteaters. Interestingly, chitin-based substrates play a pivotal role in the anteater’s diet, especially in their consumption of ants and termites. Anteaters possess a unique adaptation for chitin digestion. Chitin, a robust polysaccharide found in insect exoskeletons, provides structural support to insects and constitutes a significant portion of their diet. Specialized digestive enzymes in anteaters efficiently break down chitin, allowing them to extract vital nutrients from their insect prey (46, 48). The primary role of chitin and its derivatives in the anteater diet centers around enhancing gastrointestinal function and optimizing nutrient utilization. Chitin supplementation has emerged as a potential strategy to improve the gastrointestinal health of anteaters. By supporting normal microbiota, promoting fermentation, and influencing transit time and mineral absorption, chitin plays a crucial role. This significance is particularly pronounced for anteaters, which lack gastric acid secretion and rely on chitin-containing insects like ants and termites (47). Beyond anteater nutrition, chitin has also been investigated as a prebiotic in animal feed. Research indicates that chitin can foster the growth of beneficial gut microorganisms, ultimately enhancing overall animal health and productivity (49). We investigated the metabolic diversity and responses of various anteater clostridial isolates to chitin-based substrates. All tested clostridia were able to grow in the presence of chitin, cellulose, NAG, and glucose but varied in metabolite production. If we consider the ability of some clostridia to grow even in the basic API 20A medium, *Pae. sordellii* grew best on chitin from all tested species. In 2016, *Paeniclostridium* was proposed as a new genus (50); therefore, species belonging to *Paeniclostridium* spp. have not been studied extensively. *Pae. sordellii*, reclassified *Clostridium sordellii*, is quite well known for its pathogenicity to mammals (20, 51). On the other hand, it is a normal part of the human and animal intestinal microbiota (17, 19). However, information on its ability to degrade complex substrates such as chitin and its derivatives is lacking. Even more interesting was its inability to grow and produce metabolites in the presence of chitosan. As with the other clostridia tested, the production of metabolites was significantly lower compared to the other substrates, sometimes essentially zero. It was also confirmed that the addition of chitosan influenced the number of detected cells, when in the case of *Pae. sordellii* was completely inhibited. *P. bifermentans* and *C. colicanis* were also sensitive to chitosan. Chitosan is known for its antimicrobial effects (52–54). According to our findings, this effect is genus- and strain-specific for clostridia, and further research is desirable.

## 5 Conclusion

Microorganisms use a variety of mechanisms to regulate their abundance in the microbiota. Their growth and metabolic activity on different substrates provide insights into their potential application. However, it also raises the demand to explain these mechanisms at the genomic level. The intestinal microbiota is a complex system where interactions such as cross-feeding, nutrient competition, or antimicrobial activity are essential. The tested clostridial species, often classified as opportunistic pathogens originating from anteaters, showed diverse fermentation profiles and potential. They were able to grow in the presence of chitin with utilization potential. However, the antimicrobial effect of the chitosan derivative can ultimately regulate their numbers in the microbiota, which could be desirable to reduce their possible pathogenicity.

## Data Availability

The data presented in the study are deposited in the https://www.ncbi.nlm.nih.gov/genbank/ repository, accession numbers: PQ573793, PQ573580, PQ573579, PQ573794, PQ573795, and PQ573511.
